# EZH2 Exacerbates Breast Cancer by Methylating and Activating STAT3 Directly

**DOI:** 10.7150/jca.50675

**Published:** 2021-06-26

**Authors:** Yi Zhao, Zhansheng Hu, Jincheng Li, Tingyan Hu

**Affiliations:** The First Affiliated Hospital of Jinzhou Medical University.

**Keywords:** Breast cancer, EZH2, STAT3, Methylation

## Abstract

Breast cancer is one of the most common causes of female death globally. Numerous clinical drugs for breast cancer have been developed, but the unsatisfactory, inevitable side effects and drug resistance are the emerging threatens. Therefore, it is necessary to investigate the comprehensive mechanism of breast cancer. Enhancer of zeste homolog 2 (EZH2) is a candidate oncogenic driver in diverse cancers, such as breast cancer. The canonical role of EZH2 has been vastly investigated, but the non-canonical function of EZH2 in breast cancer remains unclear. Here, we demonstrated that EZH2 exacerbated breast cancer in non-canonical manner by methylating STAT3. EZH2 over-expressed in breast cancer patients and regulated STAT3 post-transcriptionally according to TCGA datasets. Chemical and genetic inhibition of EZH2 impeded proliferation and migration of breast cancer cells, which may be partially rescued by STAT3 over-expression. EZH2 physically interacted with STAT3 and methylated STAT3 directly, resulting in increased nuclear localization and chromatin of STAT3. Furthermore, the mutation of STAT3 methylation site, targeted by EZH2, impeded the transcriptional activity of STAT3. Eventually, disturbed STAT3 methylation by EZH2 in animal model showed decreased breast cancer growth. These data confirm that EZH2 exacerbates breast cancer by methylating STAT3 directly, and thus providing a promising therapeutic target for breast cancer.

## Introduction

Breast cancer is the most common cancer and the primary cause of mortality due to cancer in female around the world. In 2012, 1.68 million and 0.53 million of incidence and death caused by breast cancer respectively were reported in developing countries 1. Accumulating studies have been performed to get better insights into the molecular basis of the malignant condition. As a result, numerous potential clinical drugs for breast cancer have been developed and contribute much to the improved prognosis of patients 2. Nonetheless, the unsatisfactory and inevitable side effects as well as emerging drug resistance of clinical treatment underpins the necessity of comprehensive mechanistic insights into its pathogenesis, and thereof providing us with novel and specific targets and candidate drugs to circumvent those effects.

A large body of studies have manifested that EZH2 plays the pivotal role in breast cancer 3,4. As the indispensable methyltransferase core of PRC2, EZH2 methylates histone H3 on lysine 27 with the help of several other subunits. As a result, the expressions of target genes were silenced. It has been vastly shown that EZH2 is increased in breast cancer and aggravates breast cancer by regulating various genes expression in canonical manners, such as FOXC1, SOX4, CDKN2A, et al. 3,5,6. Notably, EZH2 is capable of fine-tuning expression of genes via non-canonical manners. In prostatic cancer, EZH2 promotes cancer progression by up-regulating gene expression which is aided by androgen receptor 7. Similar effects of EZH2 were shown in glioblastoma 8. Although the canonical role of EZH2 in breast cancer has been vastly investigated, the non-canonical counterpart of EZH2 in this condition is much scarcely reported.

Signal transducer and activator of transcription 3 (STAT3) is a transcriptional factor ectopically expressed in various cancer types including breast cancer 9. STAT3 exacerbates breast cancer via IL6/JAK/STAT3, EGFR/VEGFR signaling, et al. Consequently, the cancer-aggravating downstream targets, e.g. Bcl2, CCND1, MMPs, et al., are robustly up-regulated which results in cancer progression 9. Recently, Eunhee Kim demonstrated that EZH2 exacerbated glioblastoma by directly methylating STAT3 8. Therefore, whether EZH2 takes part in the cancer-promoting role of STAT3 in breast cancer remains to be clarified.

In the presented study, we showed for the first time that EZH2 exacerbates breast cancer in non-canonical manner via STAT3-mediated signalings. EZH2 promotes proliferation and migration of breast cancer cells by methylating STAT3. Mechanistically, the nuclear localization, chromatin binding as well as trans-acting activity of STAT3 is reinforced by the methylation. As a result, the expression of downstream targets is up-regulated. By contrast, the mutation of methylation site of STAT3 by EZH2 abolished those effects. Our results provide the direct evidence of the implication of non-canonical functions of EZH2 in breast cancer.

## Material and methods

### TCGA data and human breast cancer tissue staining source

All the TCGA data was obtained from and analyzed by GEPIA dataset (http://gepia.cancer-pku.cn/). Normal and cancerous breast tissue staining sections were obtained from human protein atlas dataset (http://www.proteinatlas.org/). Survival data was obtained from and analyzed by Kaplan-Meier plotter dataset (http://www.kmplot.com/).

### Cell culture

Human breast cancer cell line MCF-7, MDA-MB-231 and 4T1 cells were purchased from FDCC (Shanghai, China). Cells were cultured in DMEM (High glucose) supplemented with 10% fetal bovine serum and 1% penicillin/streptomycin. Cells were passaged when the confluence is around 90~100%. 0.5% trypsin-EDTA is applied to obtain single cell suspension and recovered by complete medium.

### Cell number counting

Cells were seeded to each well at 1×10^5^ per well. After synchronizing by starvation with serum-depleted medium for 24 hr, cells were treated with 5 μM GSK126 (MCE, New Jersey, USA) for 48 hr. Thereafter, cells were rinsed with PBS followed by trypsin treatment in humidified incubator. The complete medium was used to stop digestion followed by gently pipetting. Lastly, cell number was counted with hematocytometer under microscope.

### MTS assay

Cells were seeded into 96-well plates. After synchronizing by starvation with serum-depleted medium for 24 hr, cells were treated with 5 µM GSK126 for 48 hr. 20 µl MTS reagent (Biovision, Milpitas, USA) was added followed by incubation at 37 °C for 3 hr. Thereafter, the absorbance was determined with spectrometer.

### Scratch-healing assay

Cells were seeded on the plates and cultured until the confluency was around 90%. Then cells were treated with corresponding chemicals or lentivirus for 24h. After that, the medium was replaced with complete medium while straight gaps were introduced to each well. After 12 hr, the gaps were acquired with the inverted microscope (Leica, Wetzlar, Germany) and analyzed with Image J software.

### Cell infection with overexpression and shRNA lentivirus

Cells were starved overnight. The medium was replaced with fresh medium. Later, 1 μl overexpression or shRNA lentivirus was added to the medium. The EZH2 shRNA clones in the pLKO.1-puro vector were used: EZH2 (TRCN0000040075). The shRNA clones were obtained from Sigma-Aldrich. Plasmids used in this study were constructed by BGI (Beijing, China). Meanwhile, polybrene was added to improve the infection efficacy. The expression of target genes was showed by immunoblotting and qPCR.

### Total RNA extraction, reverse transcription, and quantitative real-time PCR

Total RNA was extracted with RNA extraction kit (BioTeke, Beijing, China) according to the manufacturer's instruction. RNA was reversely transcribed into cDNA using reverse transcription kit (Thermo Fisher Scientific Corp, Waltham, USA) according to the manufacturer's instruction. Expression of mRNA was analyzed with SYBR Green PCR master mix (Transgene, Beijing, China) according to the manufacturer's instruction. 18s was used as the internal control. The expression level was normalized to control group. Primers in this study were as following: EZH2 (Sense: 5'-TCGAGCTCCTCTGAAGCAAA-3'; Antisense: 5'-AGTATCCACATCCTCAGCGG-3'), STAT3 (Sense: 5'-AAAGCAGCAAAGAAGGAGGC-3', Anti: 5'-CTGGCCGACAATACTTTCCG-3').

### Co-immunoprecipitation (CoIP)

Total protein was extracted with immunoprecipitation lysis buffer (Solarbio life science, Beijing, China). After centrifuge, the supernatant was kept and mixed with anti-EZH2 (Cell signaling technology, Danvers, USA)/anti-STAT3 (Cell signaling technology, Danvers, USA)/anti-methylated lysine antibody (Abcam, Cambridge, USA) for 8 hrs. Then Protein A/G-coated agarose (Thermo Fisher Scientific Corp, Waltham, USA) was added to the mix and incubated at 4°C for 4 hrs. Lastly, the obtained mix was washed and de-natured with loading buffer followed by SDS-PAGE gel resolving.

### Chromatin immunoprecipitation (ChIP)

ChIP assay was performed as previously described 3. Briefly, cells were cross-linked with 1% formaldehyde and the chromatin was fragmented with sonication. 200-500bp DNA fragments were used. The fragments were co-precipitated with specific antibody for 8 hr and the obtained complex was re-precipitated with Protein A/G-coated magnetic beads (Thermo Fisher Scientific Corp, Waltham, USA) for 4 hr according to manufacturer's instruction. After reversely cross-linking by shaking at 65 °C for 2 hr, the product was amplified. The results was normalized to control group. Primers in this study were as following: CCND1 (Sense: 5'-AACTTGCACAGGGGTTGTGT-3'; Antisense: 5'-GAGACCACGAGAAGGGGTGACTG-3'), MMP2 (Sense: 5'-TGTTCCCTAAAACATTCCCC-3'; Anti: 5'-GTCTCTGAGGAATGTCTTCT-3').

### Dual luciferase reporter assay

Dual luciferase assay kit was purchased from Promega (CA, USA) and performed according to the manufacturer's instruction. Briefly, HEK293T cells were transfected with wild type/mutant STAT3, CCND1 and MMP2 promoter luciferase reporter plasmids as well as renilla control plasmids as described above. After 24h, cells were lysed with passive lysis buffer and shaken for 1 hr. Then the OD values were determined with full-wavelength spectrometer. The relative activation of CCND1 and MMP2 promoter by wild type/mutant STAT3 was showed as the OD value of luciferase to renilla.

### Total protein extraction and immunoblotting

Total protein was extracted using tissue lysis buffer (Solarbio life science, Beijing, China). Samples were quantitated and then treated with loading buffer. Each sample was loaded to and resolved by 10% SDS-PAGE gels. And protein was transferred to PVDF membranes followed by blocking. Then, the membranes were incubated with the primary antibody against EZH2 (Cell signaling technology, Danvers, USA), STAT3 (Cell signaling technology, Danvers, USA), and β-actin (Santa Cruz, Dallas, USA) as well as secondary antibody. The blots were visualized with ECL detection kit (Thermo Fisher Scientific Corp, Waltham, USA) and analyzed with Image J. GAPDH was used as internal control. The results were normalized to control group.

### Immunofluorescence staining

Cells seeded on cover glasses were rinsed with PBS and fixed with 4% paraformaldehyde. The cells were penetrated with penetration buffer (0.1% TritonX-100) and blocked with 1% BSA. The primary antibody against EZH2 (Cell signaling technology, Danvers, USA) and STAT3 (Cell signaling technology, Danvers, USA) were incubated with cells overnight. Then the secondary antibodies were applied to the cells followed by sections mounting with DAPI-containing mounting agent. All images were captured using inverted microscope (Leica, Wetzlar, Germany).

### Modeling breast tumor model with BALB/c mice

BALB/c mice (~5-week) were obtained from the Experimental Animal Center of Military Medical Science Academy, Beijing, China. Twenty female mice (5-week) were grouped into Scramble, EZH2-shRNA, EZH2-shRNA+WT STAT3 as well as EZH2-shRNA+Mut STAT3 OE. All the procedure was performed according to previous study 10. After 4T1 mammary tumor cells infection with each lentivirus, 1.5×10^5^ infected cells suspended in 100 μl PBS were injected into the mouse mammary fat pad. Mice were kept for 4-week accompanied with normal chow and drinking water. After 4-week, mice were sacrificed followed by tumor size determination. All procedures involving experimental animals were in accordance with US National Institutes of Health Guide for the Care and Use of Laboratory Animals.

### Statistical analysis

Each experiment was repeated 3 times. Student's *t*-test was applied to show statistical significance. All results were expressed as means ± SEM. *P* < 0.05 was considered as statistically significant.

## Results

### EZH2 potentially exacerbate breast cancer via STAT3 in non-canonical manner

**Figure 1:**
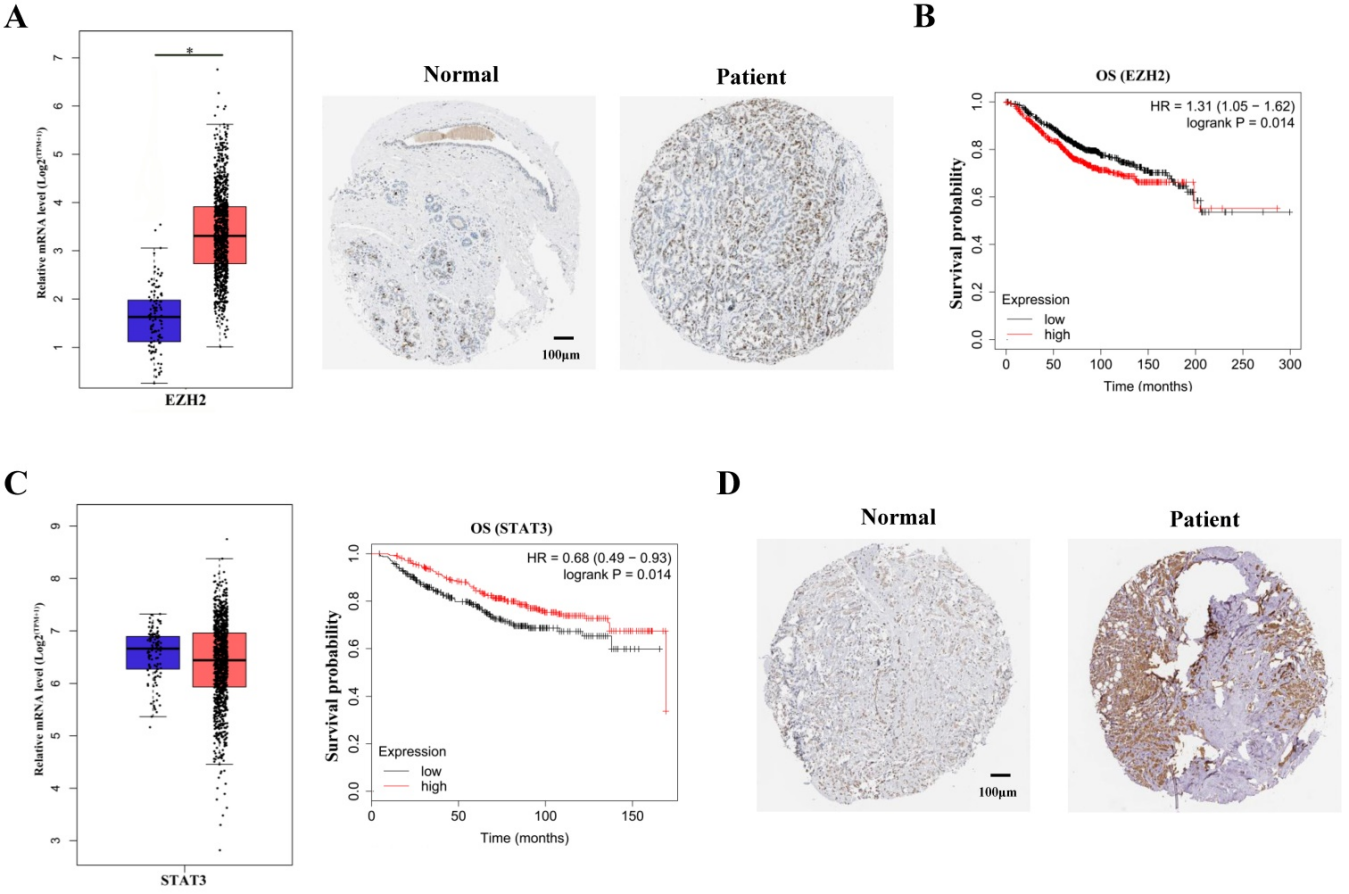
** EZH2 and STAT3 correlate with the prognosis of breast cancer. (A)** Immunohistochemical staining and mRNA expression of EZH2 in normal cohorts and patients. Samples number of normal cohorts = 1085, samples number of patients = 112. **(B)** Kaplan-Meier survival curve of EZH2 within normal cohorts and patients. Samples number of overall survival (OS) of EZH2 = 1402. **(C)** Kaplan-Meier survival curve and mRNA expression of STAT3 in normal cohorts and patients. Samples number of normal cohorts = 1085, samples number of patients = 112. Samples number of OS of STAT3 = 626. **(D)** Immunohistochemical staining in normal cohorts and patients. Scale bar = 100 µm. P value. * P<0.05, ** P<0.01.

It has been vastly reported that EZH2 expression was significantly upregulated in breast cancer patients 11. The upregulation of EZH2 expression were shown by TCGA data mining and immunohistochemical staining of breast cancer tissues (Figure 1A). In accordance with previous studies, survival analysis showed that EZH2 negatively correlated with the survival probability in breast cancer patients (Figure 1B). To manifest the potential regulation of STAT3 by EZH2, STAT3 expression was mined in TCGA database. Though EZH2 was capable of regulating gene expression in PRC2-dependent manner, STAT3 mRNA showed no difference between normal cohorts and breast cancer patients (Figure 1C, left panel). It is indicated that EZH2 might regulated STAT3 post-transcriptionally. Besides, although STAT3 mRNA showed no differenence between normal cohorts and patients, STAT3 mRNA positively correlated with the survival probability of breast cancer patients (Figure 1C, right panel). This contradicted previous studies which reported that STAT3 exacerbated breast cancer via various signaling 9,12. Since the post-transcriptional modification of STAT3, e.g. phosphorylation, was critical for its function, we reasoned that the post-transcriptional rather than the transcriptional modification of of STAT3 was implicated in EZH2-mediated progression of breast cancer^13^. In fact, immunohistocheical staining showed that STAT3 was highly expressed in patients tissue and predminantly located within nuclei which indicated its high activity in breast cancer (Figure 1D). These results suggested that EZH2 might regulate STAT3 in post-transcriptional manner rather than the canonical manner to aggravate breast cancer.

### EZH2 promotes proliferation and migration of breast cancer cells through STAT3

**Figure 2:**
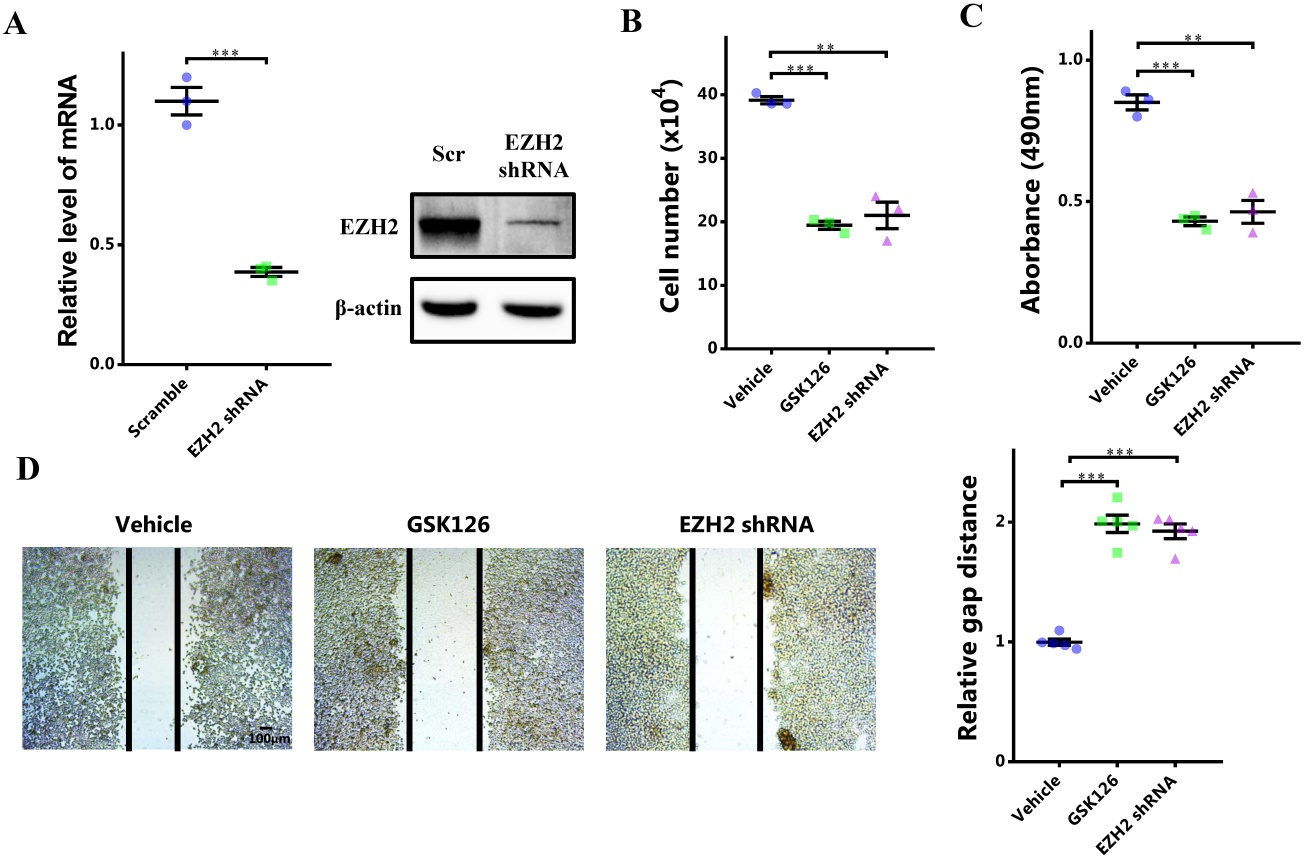

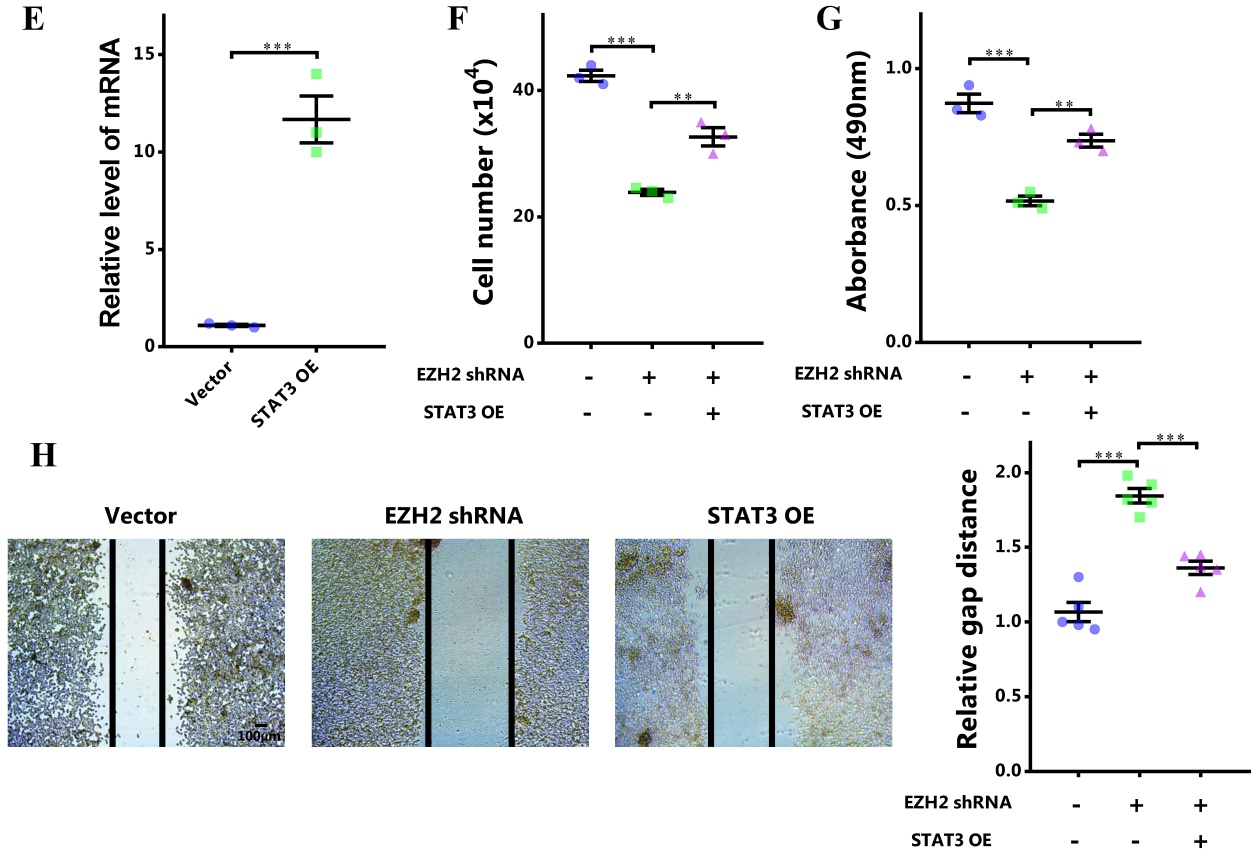
** STAT3 rescues impaired proliferation and migration resulting from EZH2 inhibition. (A and E)** mRNA expression of EZH2 and STAT3 after EZH2 knockdown and STAT3 overexpression. **(B)** Cell number counting after GSK126 and EZH2 shRNA challenge. **(C)** MTS assay of cells after GSK126 and EZH2 shRNA challenge. **(D)** Scratch-healing assay after GSK126 and EZH2 shRNA challenge. n = 5. Cell number counting **(F)**, MTS assay **(G)**, and scratch-healing assay **(H)** of EZH2-inhibited cells which are rescued by STAT3. Results are represented as mean ± SEM, n = 3, *** p < 0.001, ** p < 0.01. Scale bar = 100 µm.

To investigate the potential role of EZH2-STAT3 axis in breast cancer, we first manifested the role of EZH2 in breast cancer. EZH2 was inhibited by its specific inhibitor GSK126 or EZH2 shRNA. EZH2 was knocked down significantly (Figure 2A). After EZH2 inhibition, cells growth was markedly impaired (Figure 2B). Cell viability was also decreased (Figure 2C). Meanwhile, cell migration was decreased as well (Figure 2D). Therefore, EZH2 was capabled of promoting proliferation and migration of breast cancer cells. Nonetheless, it was not clear that whether STAT3 was involved in this signaling or not. We explored this by 'rescuing' STAT3. STAT3 was upregulated significantly using lentivirus infection (Figure 2E). Surprisingly, it was shown that the impaired cell proliferation and migration by EZH2 inhibition was rescued by STAT3 overexpression (Figure 2F, 2G and 2H). These results showed that EZH2 promotes cell proliferation and migration in breast cancer through STAT3.

### EZH2 binds to and methylates STAT3 directly

**Figure 3:**
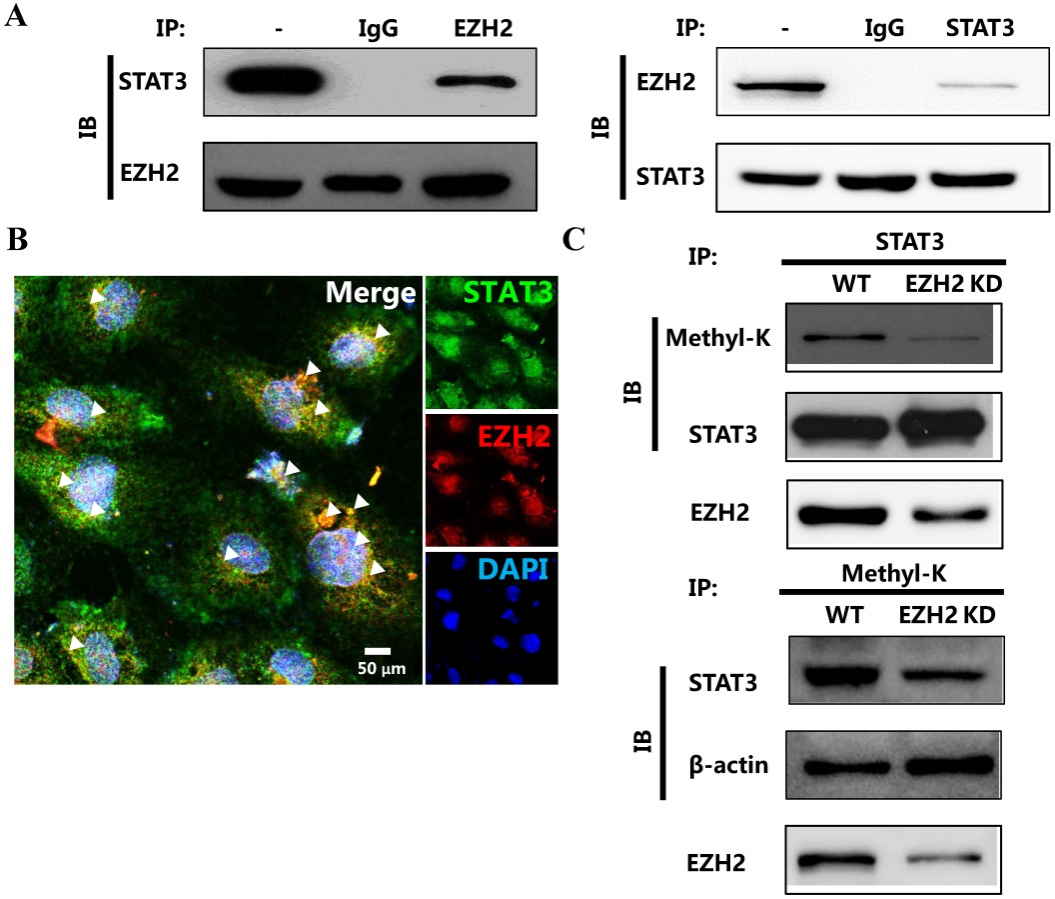
** EZH2 physically binds to and methylates STAT3.** Immunoprecipitation **(A)** and representative immunofluorescence **(B)** of EZH2 and STAT3 showing the interaction between STAT3 and EZH2. **(C)** Immunoprecipitation showing STAT3 methylation by EZH2. White arrows indicates the reciprocal binding between STAT3 and EZH2. Scale bar = 100 µm.

Recently, Eunhee Kim et al reported that EZH2 was capable of binding to and methylating STAT3 in glioblastoma 8. We deduced that EZH2 played the similar role in breast cancer. We first determined the physical interaction between EZH2 and STAT3. Apparently, EZH2 reciprocally bound to STAT3 in breast cancer cells (Figure 3A and 3B). We then determined whether EZH2 was capable of methylating STAT3 through this interaction. The results showed that lysine on STAT3 was methylated. However, this methylation was abolished by EZH2 downregulation (Figure 3C). The interaction between EZH2 and STAT3 as well as the STAT3 methylation by EZH2 was also detected in another breast cancer cell line MDA-MB-231 cells as well (Figure S2). Thus, EZH2 physically bound to and directly methylated STAT3.

### The methylation of STAT3 by EZH2 promotes its nuclear retention and transcriptional activity

**Figure 4:**
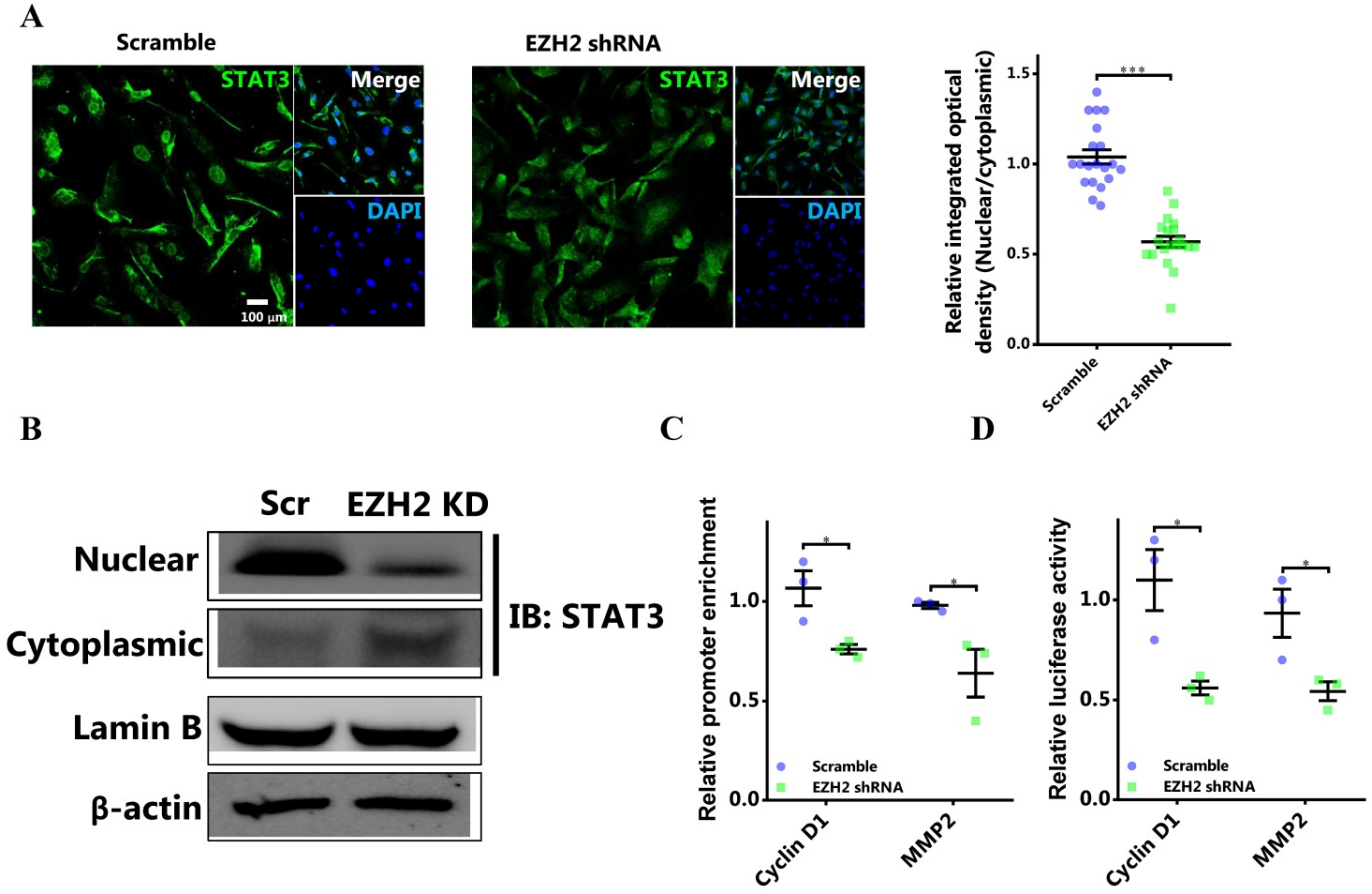
** EZH2 inhibition decreases the nuclear localization and transcriptional activity of STAT3. (A)** Representative immunofluorescence and the corresponding statistics of STAT3 after EZH2 knockdown. n = 20. **(B)** Immunoblotting of nuclear and cytoplasmic STAT3 in both Scramble and EZH2 knockdown groups, experiments were done in triplicate. **(C)** DNA binding of STAT3 to promoters of target genes. **(D)** Luciferase assay of STAT3 activating downstream target genes. Results are represented as mean ± SEM, n = 3, *** p < 0.001, * p < 0.05. Scale bar = 100 µm.

As EZH2 bound to and methylated STAT3, we determined the effect of STAT3 methylation by EZH2 on the activity of STAT3. It was shown that knocking down EZH2 could significantly decreased nuclear retention of STAT3 in breast cancer cells (Figure 4A and 4B). Therefore, STAT3 methylation by EZH2 was critical for the nuclear localization in breast cancer cells. Next, we aimed to investigate the functional implication of STAT3 methylation by EZH2. The results showed that EZH2 downregulation significantly decreased STAT3 binding to the promoter of target genes including CCND1 and MMP2 (Figure 4C). In accordance, promoter activation by STAT3 was markedly decreased by knocking down EZH2 (Figure 4D). Also, the similar results were detected in MDA-MB-231 cells (Figure S3).

Thus, EZH2 promoted nuclear retention and increased the activity of STAT3 by direct methylation.

### Blocking STAT3 methylation by EZH2 impedes nuclear retention and transcriptional activity of STAT3

**Figure 5:**
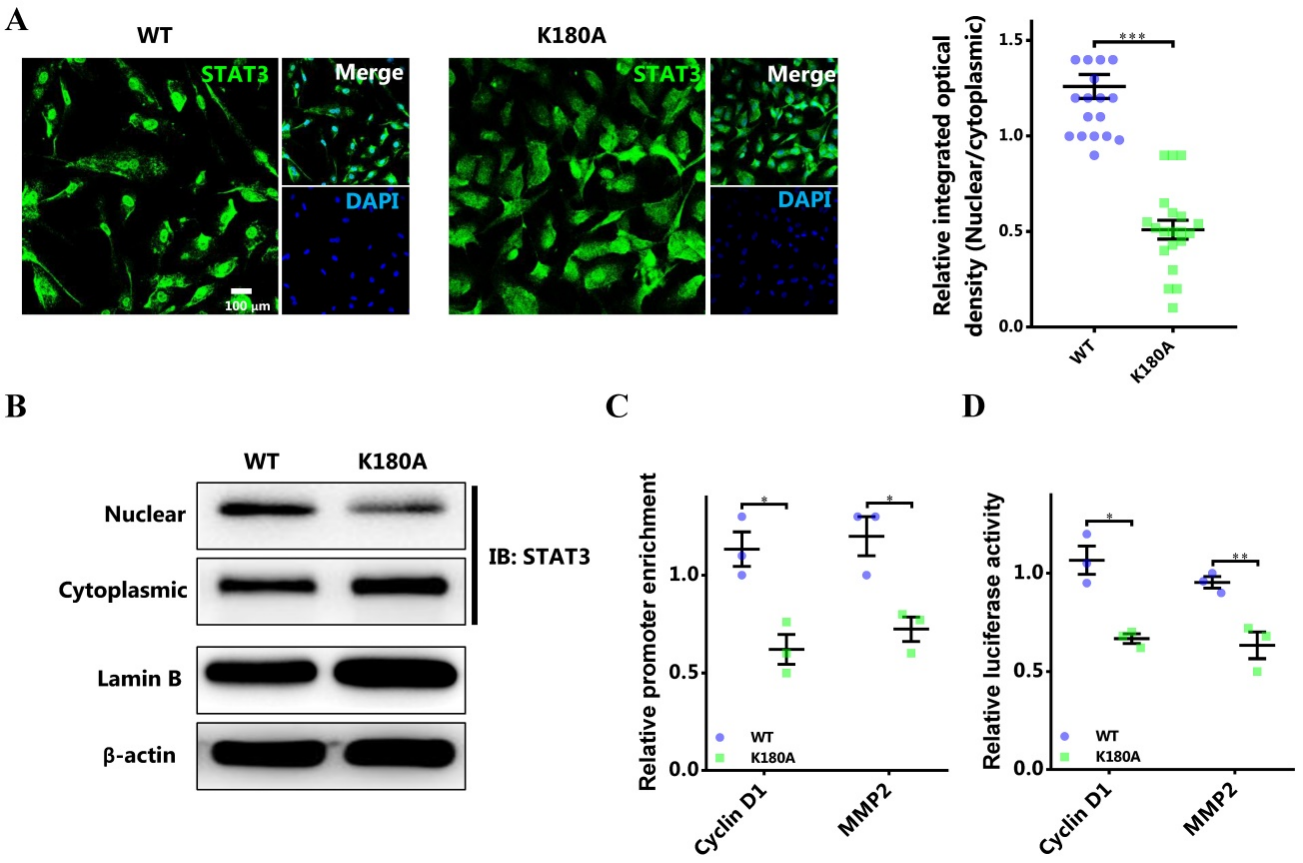

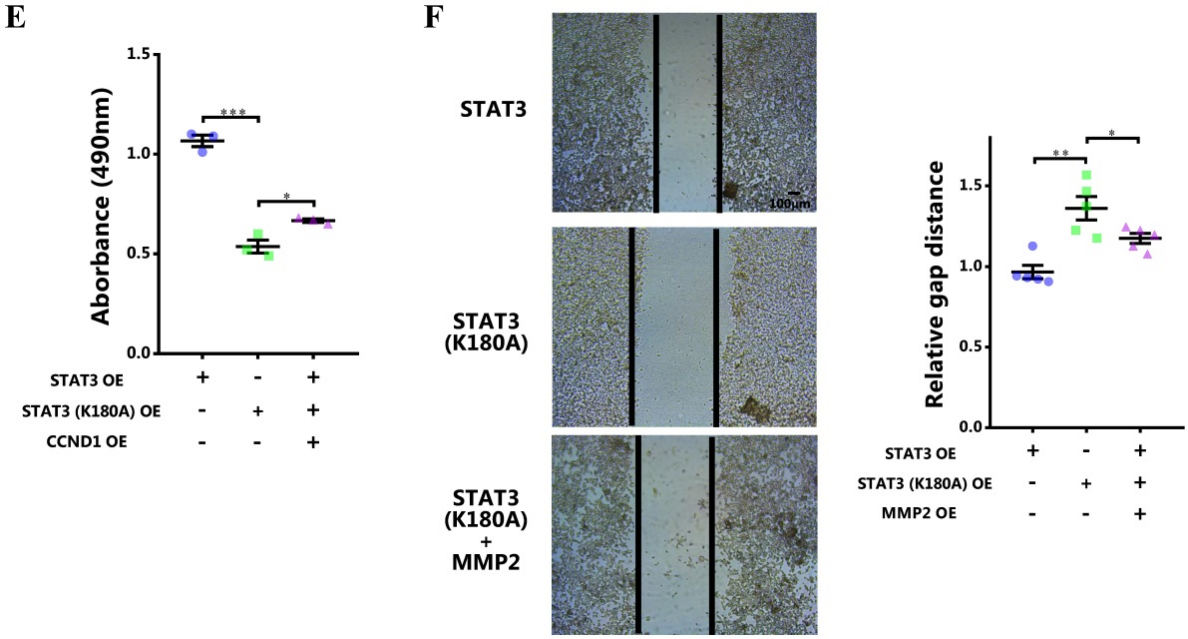
** Abolishing STAT3 methylation by EZH2 impairs its nuclear localization and transcriptional activity. (A)** Representative immunofluorescence and statistics of STAT3 in both WT and mutant cells. n = 20. **(B)** Immunoblotting of nuclear and cytoplasmic STAT3 in both WT and mutant cells. **(C)** DNA binding of STAT3 to promoters of target genes. **(D)** Luciferase assay of STAT3 activating downstream target genes. **(E)** MTS assay and **(F)** scratch-healing of cells challenged with WT or mutant STAT3 rescuing with CCND1 and MMP2 respectively. Results are represented as mean ± SEM, n = 3 or 5, *** p < 0.001, ** p < 0.01, * p < 0.05. Scale bar = 100 µm.

To further certify the functional role of STAT3 methylation by EZH2 in breast cancer, the lysine site methylated by EZH2 in STAT3 was mutated 8. The results showed that the nuclear localization of mutant STAT3 was significantly impeded (Figure 5A and 5B). Meanwhile, the promoter binding and transcriptional activity of STAT3 on target genes was remarkably impaired (Figure 5C and 5D). Further, rescue study showed that the impaired proliferation and migration by EZH2 knockdown was mitigated by CCND1 and MMP2 overexpression respectively (Figure 5E and 5F). These results further demonstrated that STAT3 methylation by EZH2 was crucial for its nuclear localization and transcriptional activity in breast cancer.

### Blocking of STAT3 methylation by EZH2 attenuates proliferation and migration of breast cancer cells

**Figure 6:**
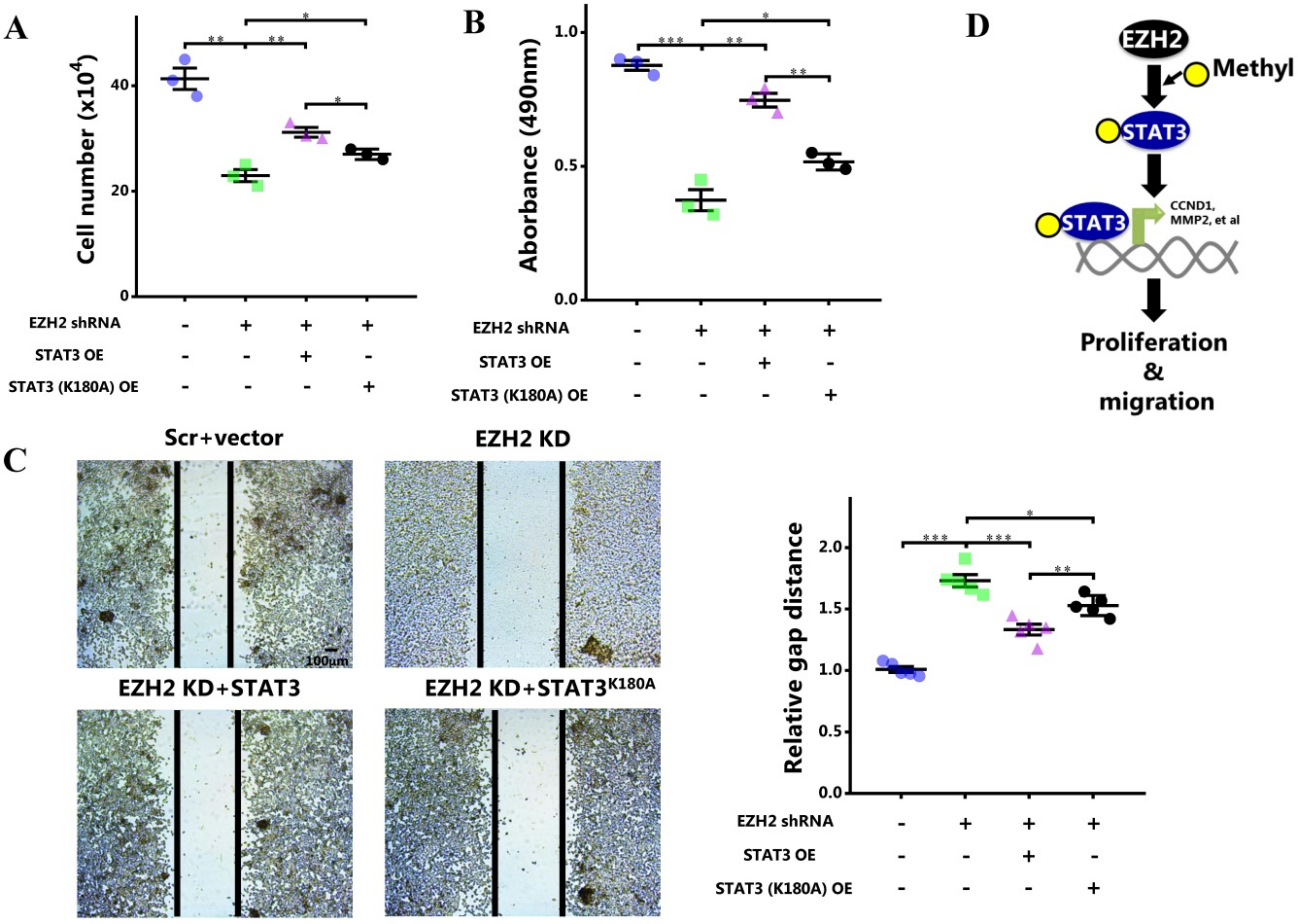
** Blocking STAT3 methylation by EZH2 inhibits proliferation and migration. (A)** Cell number counting, **(B)** MTS assay and **(C)** scratch-healing of cells challenged with EZH2 shRNA and WT or mutant STAT3. **(D)** Schematics of our hypothesis. Results are represented as mean ± SEM, n = 3, *** p < 0.001, ** p < 0.01, * p < 0.05. Scale bar = 100 µm.

As STAT3 methylation by EZH2 enhanced its activity, we eventually determined the role of STAT3 methylation in cell behaviors. It showed that although wild-type STAT3 overexpression was capable of rescuing cell growth and proliferation impaired by EZH2 knockdown, this effect was apparently attenuated when the methylation site was mutated (Figure 6A and 6B). Also, the cell migration rescued by wild-type STAT3 was abolished by methylation site mutation (Figure 6C). In MDA-MB-231 cells, the impaired proliferation and migration was mitigated by WT STAT3 which was attenuated after methylation site mutation (Figure S4). In summary, STAT3 methylation by EZH2 was critical for its nuclear retention and transcriptional activity. By methylating STAT3, EZH2 improved cell proliferation and migration of breast cancer cells. As a result, breast cancer was exacerbated (Figure 6D).

### Blocking STAT3 methylation by EZH2 mitigates breast cancer growth *in vivo*

**Figure 7:**
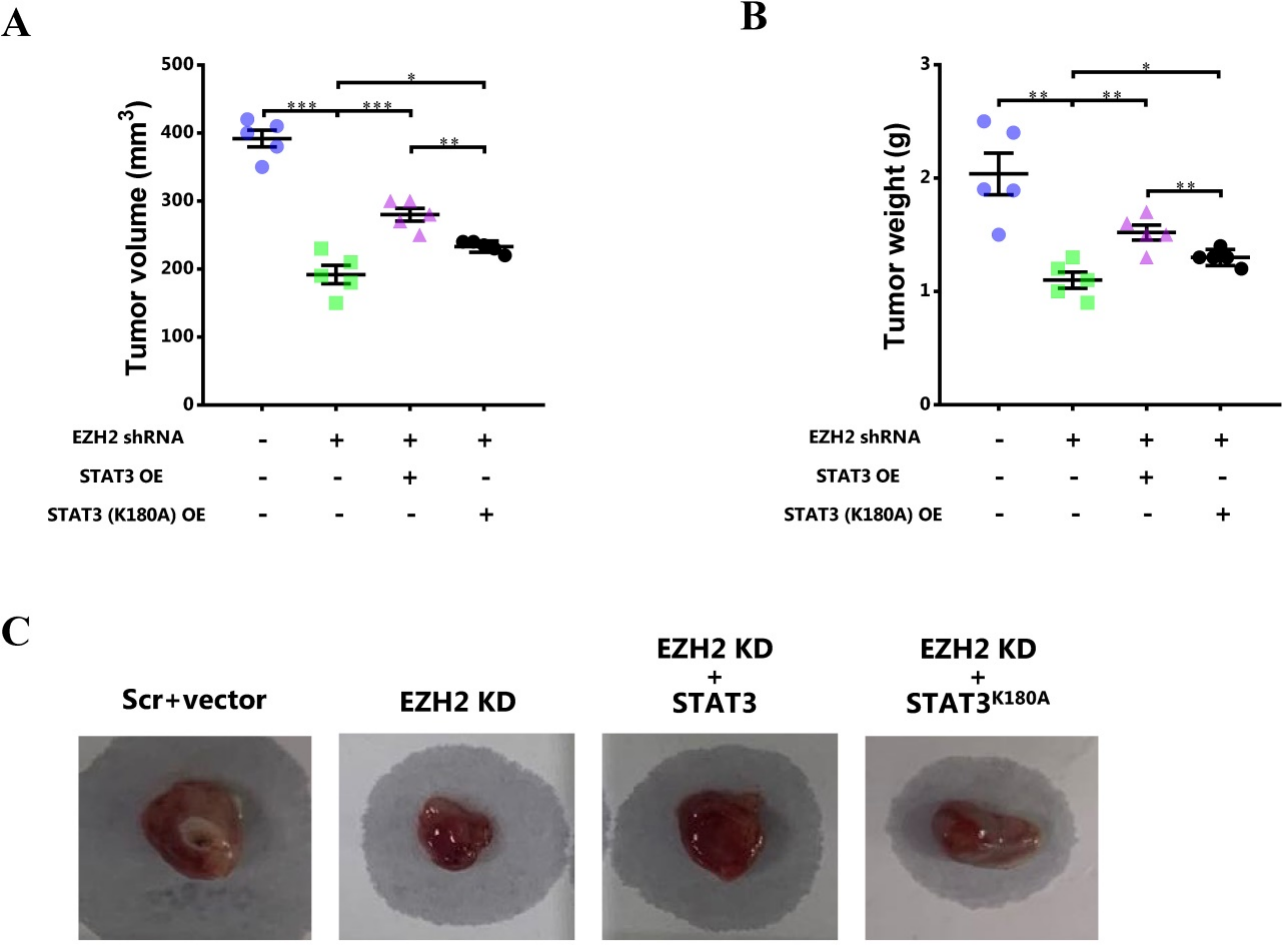
** Blocking STAT3 methylation by EZH2 mitigates breast cancer growth *in vivo*. (A)** Tumor volume and **(B)** tumor weight analyzed after 4-week. **(C)** Representative images of breast cancer in each mice. Results are represented as mean ± SEM, n = 5, *** p < 0.001, ** p < 0.01, * p < 0.05.

To further investigate the role of STAT3 methylation by EZH2 in breast cancer *in vivo*, the mouse model of breast cancer was constructed. After 4-week, the tumor size and weight was determined. It showed that the tumor size and tumor weight was obviously decreased after EZH2 knockdown while the decreased tumor size and tumor weight was recovered by WT STAT3 overexpression (Figure 7). However, mutant STAT3 attenuated the effect of EZH2 knockdown on tumor growth to less extent (Figure 7).

## Discussion

Breast cancer is the most common cause of death in women globally. Although a body of spotlight has been shed upon the pathogenesis of this fatal condition, much deeper insights into this fatal condition are urgently required. Herein, in our study, we reported that the histone methyltransferase EZH2 was capable of directly methylating the critical transcription factor STAT3. The methylation of STAT3 by EZH2 was critically important for its nuclear retention and transcriptional activity. As a result, STAT3 upregulated the expression of breast cancer-aggravating genes, such as CCND1 and MMP2, thereby enhancing the proliferation and migration of breast cancer cells.

STAT3 is closely associated with pathogenesis of various cancers by regulating the expression of critical genes for survival, proliferation, and angiogenesis, such as Bcl-2, Bcl-xL, Mcl-1, CCND1, and VEGF, which were important for survival, proliferation, and angiogenesis. However, it is not clear how STAT3 correlates with EZH2 in breast cancer. Recently, Kwang Seok Ahn et al. reported that STAT3 promoted EZH2 expression transcriptionally and thus exacerbated gastric cancer 14. Nonetheless, whether this signaling is implicated in breast cancer remains to be clarified. By contrast, EZH2 was reported to increase STAT3 phosphorylation and activation by non-canonically methylating STAT3 8. However, this has not been investigated in breast cancer. Though TCGA data mining showed that STAT3 mRNA was not altered between normal cohorts and patients, survival and protein analysis showed that STAT3 was associated with breast cancer. As breast cancer is a heterogeneous disease which could be grouped into Luminal A, Luminal B, HER2-enriched, and Triple negative subtypes, we further explored the relationship of EZH2 and STAT3 expression with survival of patients in breast cancer subtypes (Figure S1). It showed that high EZH2 expression was associated with poor survival in Luminal A cohorts while no significant involvement of EZH2 in the overall survival of Luminal B, HER-enriched, and Triple negative cohorts was detected. On the other hand, high STAT3 expression was negatively associated with the survival of the Triple negative cohorts while no significant correlation with other subtypes was detected. However, our study using both non-triple and triple negative cell lines suggested that STAT3 contributed to breast cancers with specific post-transcriptional modification. Thus, though transcriptomics was capable of providing us with valuable clues for our investigation, it was also critical to shed lights upon post-transcriptional modifications which played crucial role in protein function as well. Here, we reported for the first time that EZH2 exacerbates breast cancer by directly methylating and activating STAT3. However, whether EZH2 regulation by STAT3 signaling participates in this malignancy is not clear and this is the limitation of the presented study.

As the core methyltransferase of PRC2, EZH2 canonically modifies histone H3 lysine 27 with di/tri-methyl groups. Resultantly, genes associated with specific cell lineages including HOX, BMP, and NANOG are orchestrated 15. Pathologically, upregulated EZH2 in varied cancers including breast cancer was reported 11. Enhanced EZH2 expression indicates the poor prognosis of different cancers 16. Specifically, upregulated EZH2 is capable of promoting proliferation and migration of breast cancer cells 17,18. Although numerous efforts have been afforded to unravel the mechanisms of EZH2 involvement in breast cancer, they focus upon the canonical function of EZH2 mostly. Given the increasing morbidity of breast cancer, novel underlying mechanisms are required to be clarified. Actually, side from the chromatin-dependent role, EZH2 takes part in the pathogenesis of different cancers in non-canonical manner 19. Interestingly, it is also reported that EZH2 directly methylates pivotal regulators and thus regulates the progression of several cancers 8,20. Though it is reported that STAT3 regulates EZH2 expression at transcriptional level, it is not certified that whether EZH2 functions through STAT3 and how it fine-tunes STAT3. Our study demonstrated that EZH2 exacerbates breast cancer through STAT3 signaling in non-canonical manner. This observation partially demonstrates the conflicting results mined from TCGA databases. Specifically, post-transcriptional modification of STAT3 by EZH2 is critical for STAT3 functions besides transcriptional regulation. However, our result did not study the potential regulation of EZH2 by STAT3 in canonical manner. We deduce that STAT3 might regulates EZH2 expression which constitutes the 'vicious cycle' with STAT3 methylation by EZH2 in breast cancer.

In conclusion, our study demonstrated that EZH2 non-canonically methylated and promoted the nuclear localization of pivotal transcriptional factor STAT3 in breast cancer. As a result, the expression of targets enhancing proliferation and migration is increased. Eventually, breast cancer is initiated or progressed.

## Supplementary Material

Supplementary figures.
