# Supplementary material for: EZH2 Exacerbates Breast Cancer by Methylating and Activating STAT3 Directly
Source: J Cancer. 2021 Jun 26;12(17):5220–30. doi: 10.7150/jca.50675 (PMC8317538; doi:10.7150/jca.50675)
Supplement: Supplementary file 1 — Supplementary figures. [file jcav12p5220s1.pdf]

Figure S1 The involvement of EZH2 and STAT3 in OS of different breast cancer subtypes

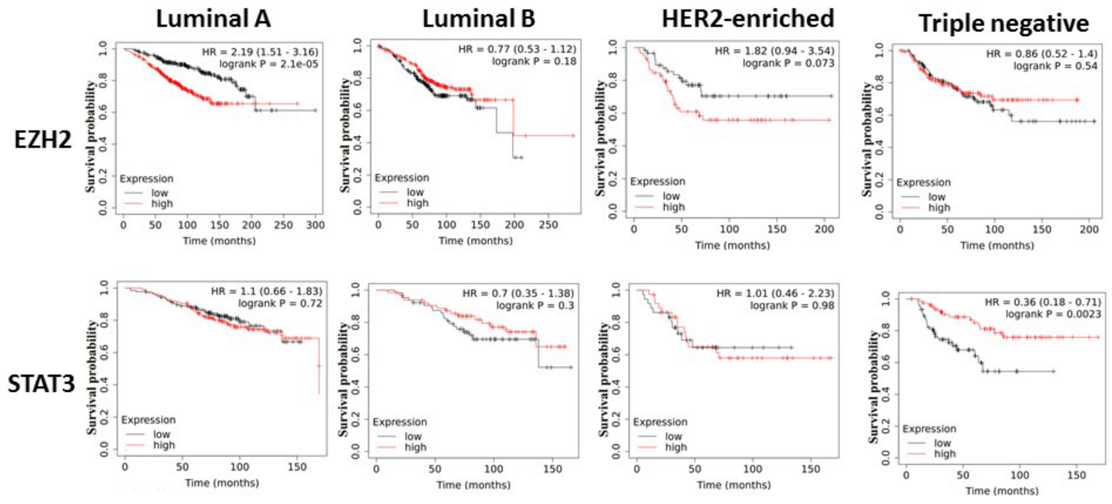

Figure S1. Correlation of EZH2 and STAT3 with the overall survival of different breast cancer subtypes. Samples number of Luminal A = 611, samples number of Luminal B = 433, samples number of HER2-enriched = 117, samples number of Triple negative = 241.

Figure S2 EZH2 methylates STAT3 in MDA-MB-231 cells

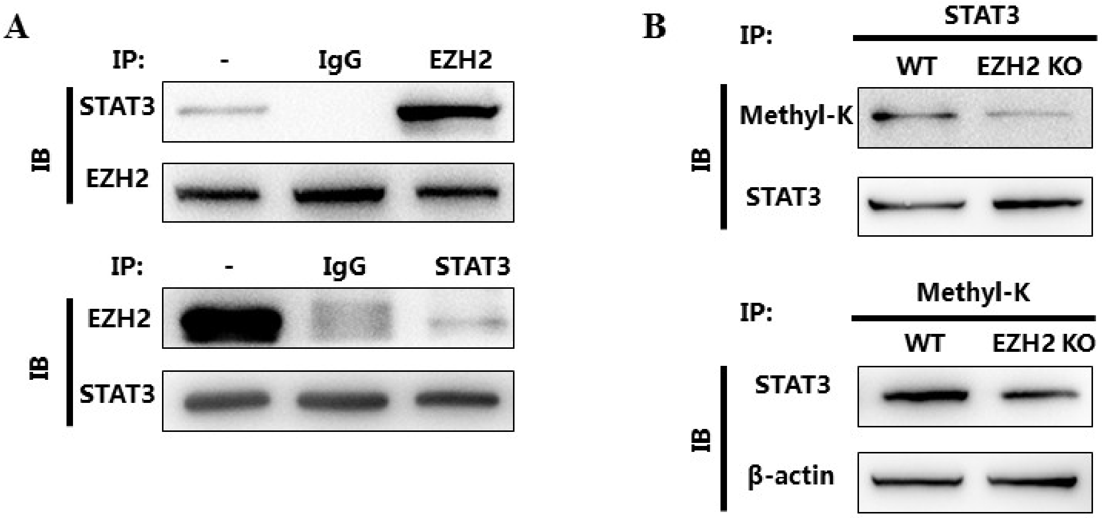

Figure S2. EZH2 physically binds to and methylates STAT3 in MDA-MB-231 cells. (A) Representative immunoprecipitation of EZH2 and STAT3 showing the interaction between STAT3 and EZH2 as well as (B) STAT3 and methylated K showing STAT3 methylation by

EZH2. Whole cell lysates are collected followed by immunoprecipitation of EZH2, STAT3 or methylated lysine respectively.

Figure S3 EZH2 increases its nuclear localization in MDA-MB-231 cells

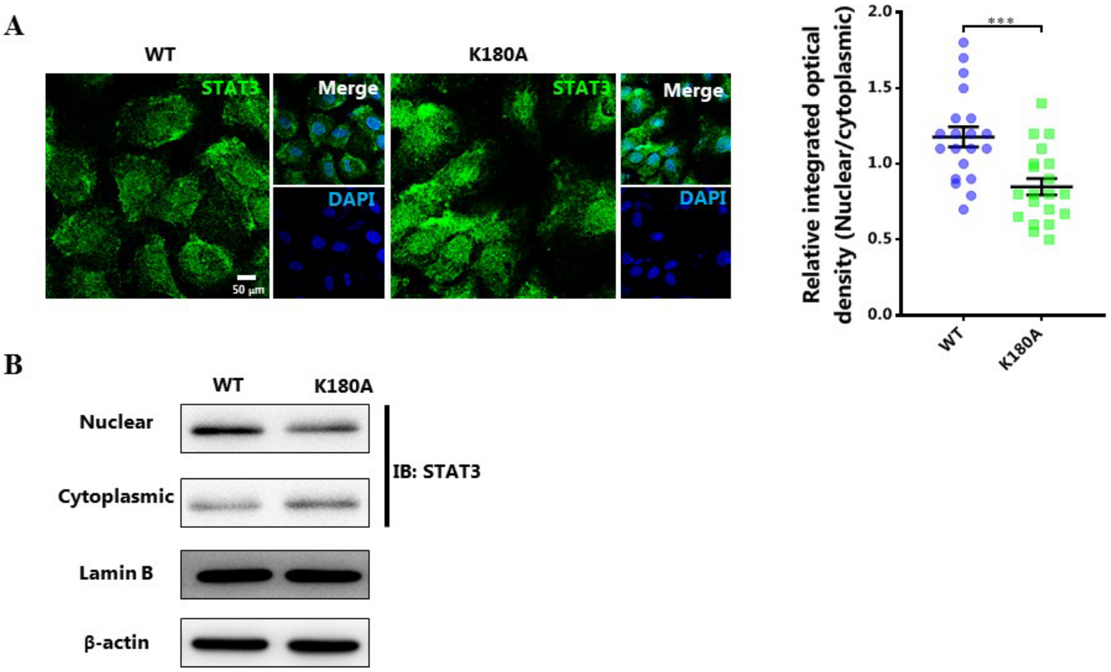

Figure S3. Abolishing STAT3 methylation by EZH2 impairs its nuclear localization in MDA-MB-231 cells. (A) Representative immunofluorescence and statistics of STAT3 in both WT and mutant cells. n = 20. After fixation, the cytoplasmic and nuclear localization

of STAT3 after methylation site mutation are visualized with inverted microscopy accompanied with IOD analysis. (C) Immunoblotting of nuclear and cytoplasmic STAT3 in both WT and mutant cells. The nuclear and cytoplasmic fraction of STAT3 are determined with or without methylation sites mutation. Results are represented as mean ± SEM, n = 20, *** $p < 0.001$. Scale bar = 50 μm.

Figure S4 Blocking the methylation of STAT3 by EZH2 inhibits proliferation and migration of MDA-MB-231 cells.

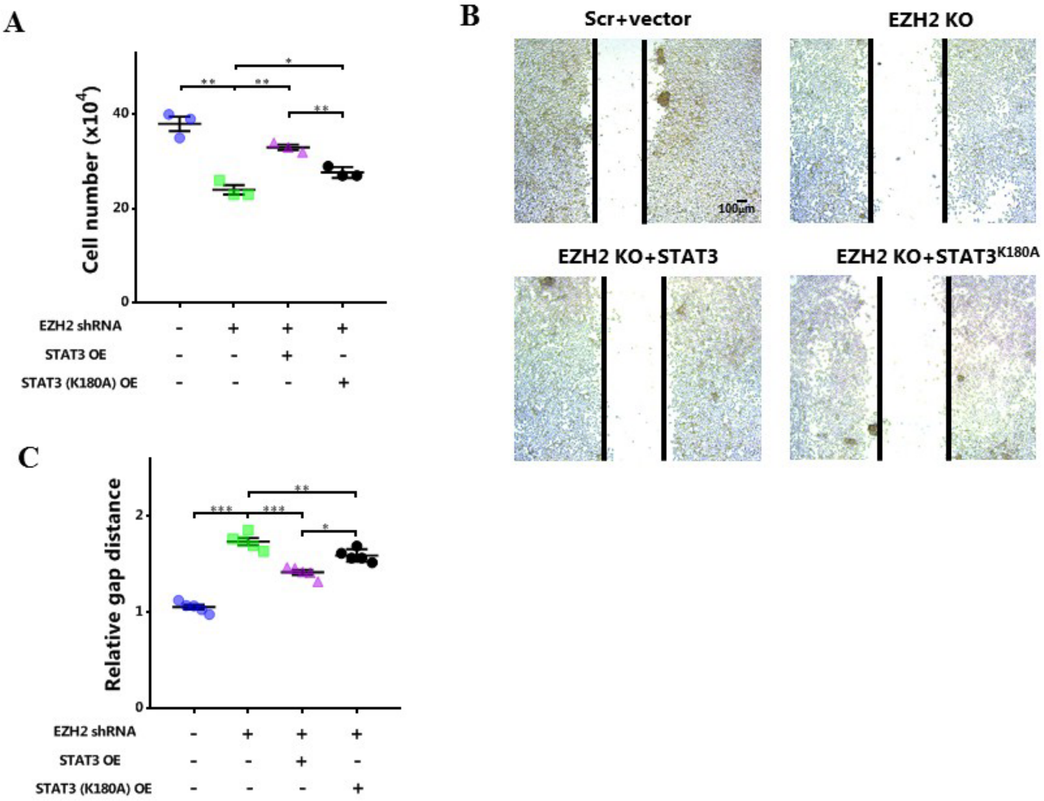

Figure S4. Blocking the methylation of STAT3 by EZH2 inhibits proliferation and migration of MDA-MB-231 cells. (A) Cell number counting. Cells are plated at the same number followed by the number counting. (B) Scratch-healing of cells challenged with EZH2 shRNA and WT or mutant STAT3 and the statistics (C). Results are represented as mean

± SEM, n = 3 or 5, *** $p < 0.001$, ** $p < 0.01$, * $p < 0.05$. Scale bar = 100 μm.
